# In Vitro Prebiotic Potential of *Opuntia humifusa* Leaf Extract and Its Active Constituent

**DOI:** 10.3390/molecules30153124

**Published:** 2025-07-25

**Authors:** Min Sung Ko, Da Bin Eom, Chung Hyeon Lee, Tae-Eun Park, Sang Jae Lee, Cheol Hyun Kim, Hui Won Moon, Seo An Lee, Kwang Woo Hwang, So-Young Park

**Affiliations:** 1 Laboratory of Pharmacognosy, College of Pharmacy, Dankook University, Cheonan 31116, Republic of Korea; dkrkekxn@naver.com (M.S.K.); uom29@naver.com (D.B.E.); cndgus1995@naver.com (C.H.L.); qkrxodms53@naver.com (T.-E.P.); 2 Laboratory of Dairy Science and Technology, College of Bio-Convergence, Dankook University, Cheonan 31116, Republic of Korea; sjlee3765@naver.com (S.J.L.); hichkim@dankook.ac.kr (C.H.K.); 3 Defense Modulation Laboratory, College of Pharmacy, Chung-Ang University, Seoul 06974, Republic of Korea; mhw3614000@gmail.com (H.W.M.); eeseoan@gmail.com (S.A.L.); khwang@cau.ac.kr (K.W.H.)

**Keywords:** *Opuntia humifusa*, *Lactobacillus paracasei* KCTC 12576 strain, prebiotic, isorhamnetin 3-O-β-D-(6-O-α-L-rhamnosyl)glucoside, standardization

## Abstract

*Opuntia humifusa* (commonly known as Cheon-nyun-cho) has traditionally been used for its antioxidant, laxative, and immune-boosting properties, but its potential prebiotic activity remains largely unexplored. In this study, we evaluated the prebiotic potential of *O. humifusa* leaf and fruit extracts by assessing their effects on the growth of four *Lactobacillus* spp. strains. Among them, *Lactobacillus paracasei* KCTC 12576 exhibited the most pronounced response to the extracts and was therefore selected for further investigation. Comparative analysis demonstrated that ethanol extracts were more effective than water extracts, and leaf extracts outperformed fruit extracts in enhancing bacterial viability. Notably, the ethanol extract of *O. humifusa* leaves showed the strongest stimulatory effect on *L. paracasei* KCTC 12576 growth. Based on extraction optimization studies, 60% ethanol was identified as the most effective solvent for obtaining bioactive compounds. HPLC analysis revealed the presence of isorhamnetin 3-O-β-D-(6-O-α-L-rhamnosyl)glucoside (**1**) as a major flavonol glycoside in the extract. A robust and validated HPLC method was established for quantification of this compound (0.33 mg/g in the 60% ethanol extract of the leaves), supporting the standardization of the extract. These findings suggest that *O. humifusa* leaf extract, particularly the 60% ethanol extract, may serve as a promising natural prebiotic ingredient for use in functional foods or synbiotic formulations.

## 1. Introduction

Prebiotics are defined as non-digestible food components that selectively stimulate the growth and/or activity of beneficial microorganisms in the host’s gastrointestinal tract, thereby contributing to host health [1]. To function as a prebiotic, a compound must resist digestion in the upper gastrointestinal tract and reach the colon intact, where it is fermented by gut microbiota. The fermentation process enhances the proliferation and activity of beneficial bacteria, such as *Lactobacillus* and *Bifidobacterium*, which are associated with improved immune function, intestinal barrier integrity, and even mental well-being [2].

Recently, the concept of prebiotics has evolved beyond merely non-digestible fibers. It now includes compounds that can exert specific health benefits through interactions with the host microbiome [3]. Of particular interest is the emerging evidence related to the gut–brain axis, which highlights the impact of gut microbial communities on neural, psychological, and behavioral processes [4]. This has broadened the scope of prebiotic research, positioning gut microbiota modulation as a promising strategy for systemic health regulation, including inflammation, immunity, and neurocognition.

Commercially available prebiotics include various types of oligosaccharides (such as fructo-oligosaccharides, galacto-oligosaccharides, and xylo-oligosaccharides), dietary fibers (including inulin, polydextrose, and psyllium husk), and sugar derivatives such as lacto-sucrose. Among these, fructo-oligosaccharides, in particular, have been shown to support intestinal homeostasis and alleviate symptoms of functional bowel disorders [5,6]. In recent years, attention has also turned to plant-derived flavonoids and glycosylated phenolics for their dual role as antioxidants and potential prebiotic modulators [7].

*Opuntia humifusa*, commonly known as Cheon-nyun-cho in Korea, is a member of the Cactaceae family and is traditionally consumed for its health-promoting properties. It is rich in bioactive compounds such as flavonoids, polysaccharides, dietary fiber, vitamin C, calcium, magnesium, and β-carotene. Several studies have reported its antioxidant, antidiabetic, anti-inflammatory, and anti-cancer effects; however, its prebiotic potential remains largely unexplored.

In this study, we aimed to evaluate the prebiotic potential of *O. humifusa* leaf and fruit extracts by assessing their effects on the viability of *Lactobacillus paracasei* KCTC 12576, a well-characterized probiotic strain. In particular, we focused on isolating and identifying the bioactive compound(s) contributing for the observed effects, followed by their quantitative analysis using validated HPLC methods. Furthermore, optimization of the extraction conditions and standardization of the active constituent were performed to establish a basis for its potential application as a novel, natural prebiotic ingredient in functional foods or synbiotic formulations.

## 2. Results

### 2.1. Prebiotic Effects of O. humifusa Leaf and Fruit Extracts

**Figure 1:**
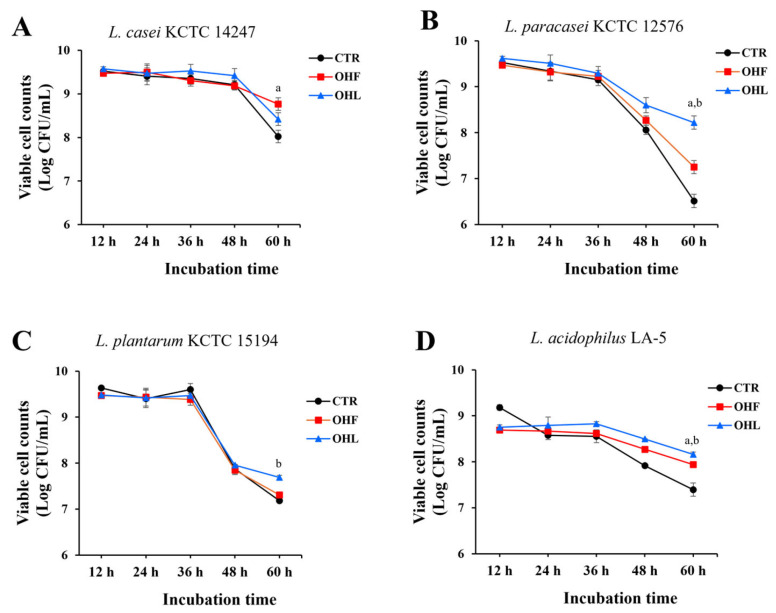
Cell viability of probiotic strains after inoculation with the ethanol extracts of *O. humifusa* leaves and fruits up to 60 h. (**A**) *L. casei* KCTC 14247, (**B**) *L. paracasei* KCTC 12576, (**C**) *L. plantarum* KCTC 15194, (**D**) *L. acidophilus* LA-5. The concentration of the ethanol extracts of *O. humifusa* leaves and fruits used in this study was 0.5 mg/mL (CTR: DMSP-treated control, OHF: *O. humifusa* fruit extract, and OHL: *O. humifusa* leaf extract). The control group was treated with 0.1% DMSO. ^a^ *p* < 0.05, *O. humifusa* fruit extract vs. DMSO-treated control; ^b^ *p* < 0.05, *O. humifusa* leaf extract vs. DMSO-treated control.

To evaluate the prebiotic effects of *O. humifusa* leaf and fruit extracts, the extracts (0.5 mg/mL) were added to MRS broth inoculated with four *Lactobacillus* species, *L. casei* KCTC 14247, *L. paracasei* KCTC 12576, *L. plantarum* KCTC 15194, and *L. acidophilus* LA-5. The cultures were incubated for up to 60 h, and viable bacterial counts were measured at 12, 24, 36, 48, and 60 h. Among the tested strains, *O. humifusa* leaf and fruit extracts notably enhanced the growth of *L. paracasei* KCTC 12576, suggesting a potential prebiotic effect (Figure 1). Specific death rates (μₑ) were also calculated for the period between 36 and 60 h. Among all tested conditions, the ethanol extract of *O. humifusa* fruits induced notable declines in *L. plantarum* KCTC 15194 (μₑ = 0.1995 h^−1^). In contrast, lower μₑ values were observed in *L. acidophilus* LA-5 treated with ethanol extracts of fruits and leaves (μₑ = 0.0648 and 0.0635 h^−1^, respectively), in *L. casei* KCTC 14247 treated with fruit ethanol extract (μₑ = 0.0517 h^−1^), and in *L. paracasei* KCTC 12576 treated with ethanol extracts of leaves. When compared with their respective controls, *L. casei* KCTC 14247 treated with fruit extract showed the largest reduction in death rate (Δμₑ = −0.0758 h^−1^), and *L. paracasei* KCTC 12576 treated with leaf extract exhibited a pronounced reduction (Δμₑ = −0.1501 h^−1^). Consequently, *L. paracasei* KCTC 12576 was selected for evaluation of the prebiotic potential of O. humifusa extracts based on its consistent growth performance, physiological stability, and suitability for the intended application.

**Figure 2:**
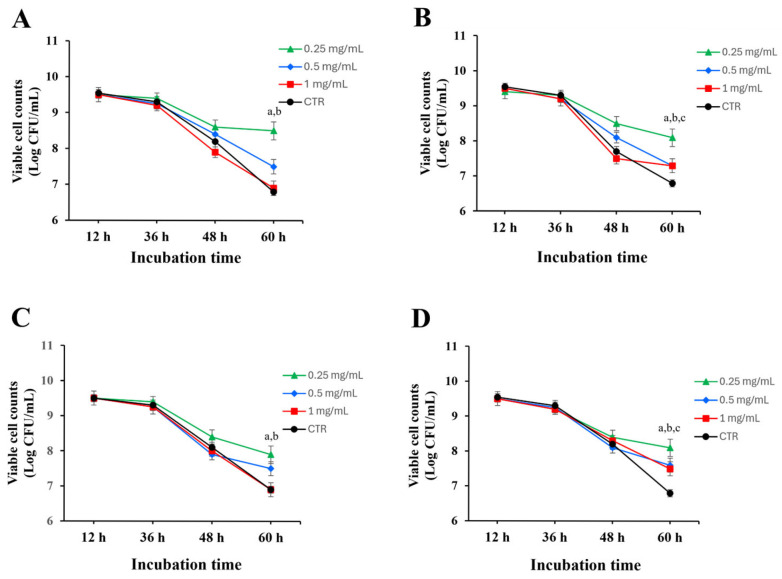
Cell viability of *L. paracasei* KCTC 12576 after inoculation with the ethanol and water extracts of *O. humifusa* leaves and fruits up to 60 h. (**A**) The ethanol extract of *O. humifusa* leaves (0.25, 0.5, and 1 mg/mL), (**B**) the water extract of *O. humifusa* leaves (0.25, 0.5, and 1 mg/mL), (**C**) the ethanol extract of *O. humifusa* fruits (0.25, 0.5, and 1 mg/mL), (**D**) the water extract of *O. humifusa* fruits (0.25, 0.5, and 1 mg/mL). The control group was treated with 0.1% DMSO. ^a^ *p* < 0.05, 0.25 mg/mL of the extract vs. DMSO-treated control; ^b^ *p* < 0.05, 0.5 mg/mL of the extract vs. DMSO-treated control; ^c^ *p* < 0.05, 1.0 mg/mL of the extract vs. DMSO-treated control.

The prebiotic was further examined by incubating *L. paracasei* KCTC 12576 with leaf and fruit extracts at concentrations of 0.25, 0.5, and 1 mg/mL. For up to 36 h, both ethanol or water extracts from leaves and fruits had minimal impact on bacterial growth, with no significant changes in cell counts compared to the control. However, at 60 h, both water and ethanol extracts at 0.25 mg/mL significantly increased the survival rate of *L. paracasei* KCTC 12576 relative to the control. Notably, ethanol extracts exhibited greater activity than water extracts, and the leaf extracts effectively enhanced the viability of *L. paracasei* KCTC 12576 than the fruit extracts, indicating their potential as prebiotic agents (Figure 2). To quantitatively assess the effect of each extract on bacterial viability, specific death rates (μₑ) were calculated for the time interval between 36 and 60 h. The highest death rates were observed in the control groups of both stem and fruit extracts (μₑ = 0.2399 h^−1^), but the ethanol extract of *O. humifusa* leaves demonstrated the highest activity. Among the extract-treated groups, the 1 mg/mL ethanol extracts of fruit and stem showed relatively high death rates (μₑ = 0.2255 and 0.2207 h^−1^, respectively), while the lowest death rates were observed in the 0.25 mg/mL stem ethanol extract (μₑ = 0.0864 h^−1^), followed by fruit water extract (0.1056 h^−1^), and stem water extract (0.1152 h^−1^). When compared with their respective controls, the 0.25 mg/mL ethanol extract of *O. humifusa* leaves exhibited the greatest reduction in specific death rate compared to the control (Δμ = 0.1535 h^−1^). The ethanol extract of *O. humifusa* leaves demonstrated the highest activity, suggesting that it is the most effective among the tested samples in promoting probiotic survival.

### 2.2. Isolation and Structural Identification of Active Compounds from O. humifusa Extract

Subsequently, the ethanol extract of *O. humifusa* leaves was used to isolate the active compounds responsible for the prebiotic effects. The leaves of *O. humifusa* were chosen due to their better activity, year-round availability, lower sugar content, and practical advantages in procurement, as fruits are only available during specific seasons.

Sugars were first removed from the leaf extracts using Amberlite resin, followed by repeated medium pressure liquid chromatography (MPLC) with a C18 stationary phase to isolate compound **1** in its pure form.

**Figure 3:**
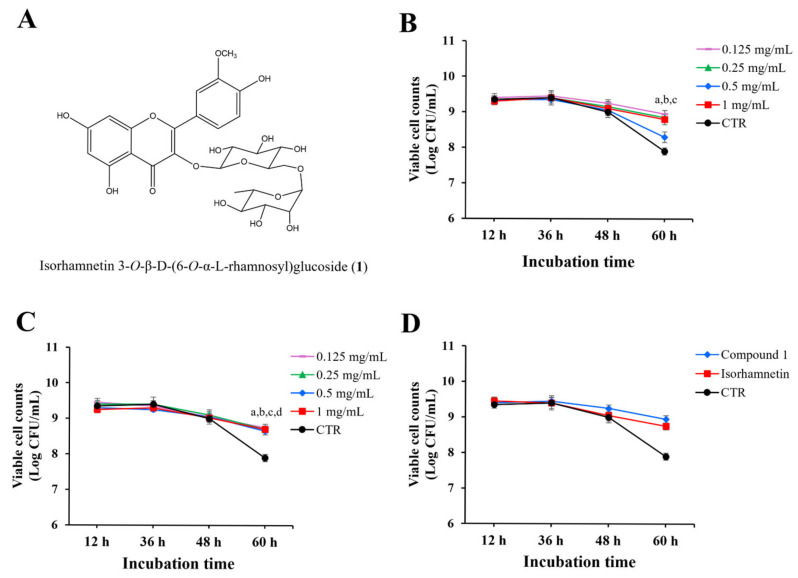
Structure of compound **1** and its prebiotic effects. (**A**) Structure of compound **1**, (**B**) cell viability of *L. paracasei* KCTC 12576 after inoculation with compound **1** up to 60 h, (**C**) cell viability of *L. paracasei* KCTC 12576 after inoculation with isorhamnetin (aglycone of compound **1**) up to 60 h, (**D**) comparison on cell viability of *L. paracasei* KCTC 12576 after inoculation with compound **1** or isorhamnetin at 0.125 mg/mL. ^a^ *p* < 0.05, 0.125 mg/mL of the compound vs. DMSO-treated control; ^b^ *p* < 0.05, 0.25 mg/mL of the compound vs. DMSO-treated control; ^c^ *p* < 0.05, 0.5 mg/mL of the compound vs. DMSO-treated control; ^d^ *p* < 0.05, 1 mg/mL of the compound vs. DMSO-treated control.

Compound **1** (Figure 3A) was obtained as a yellow powder. The ^1^H-NMR spectrum displayed five characteristic proton signals at *δ* 6.21 (d), 6.44 (d), 7.87 (d), 7.52 (d), and 6.92 (dd), confirming the presence of a flavonol skeleton. Through HMBC correlations between *δ* 5.45 (1H, d) and the C-3 carbon, it was determined that a glucoside moiety was attached at the 3-position. In the HSQC spectrum, protons corresponding to the 6-position of the glucoside were observed at *δ* 3.28 and *δ* 3.65, and further HMBC correlations established their linkage to the anomeric carbon of the rhamnosyl group. Taken together, these spectral data confirmed that compound **1** is isorhamnetin 3-O-β-D-(6-O-α-L-rhamnosyl)glucoside, a diglycosylated flavonol with two sugar moieties attached at the C-3 position [8,9].

### 2.3. Effects of Compound **1** on the Viability of L. paracasei KCTC 12576

To evaluate the effects of compound **1** on the viability of *L. paracasei* KCTC 12576, various concentrations of compound **1** were added to liquid MRS broth cultures of *L. paracasei* KCTC 12576, and cell viability was monitored for 60 h. As shown in Figure 3B, there was no significant difference in bacterial counts up to 48 h, regardless of the presence of the *O. humifusa* leaf extract. However, at 60 h, the bacterial count in the DMSO-treated control was approximately 8 log CFU/mL, while the groups treated with compound **1** showed increased bacterial counts at all concentrations tested.

To determine whether this growth-promoting effect was due to the presence of sugars or the non-glycosylated aglycone form of compound **1**, isorhamnetin, a parallel experiment was conducted using isorhamnetin alone (Figure 3C). The treatment of bacteria with various concentrations of isorhamnetin up to 48 h did not affect the number of viable cell counts compared to the control. However, at 60 h, the bacterial count in the isorhamnetin-treated samples was significantly higher than the control.

Then, the effects of compound **1** and isorhamnetin (0.125 mg/mL) on the cell counts were directly compared in Figure 3D. The cell counts treated with both compounds at 60 h incubation were significantly higher than the control, and the effect of compound **1** was slightly higher than that of isorhamnetin, but not statistically significant.

Both compound **1** and isorhamnetin significantly reduced the specific death rate (μₑ) of *L. paracasei* KCTC 12576 compared to the control (μₑ = 0.1439 h^−1^). Compound **1** at 0.125 mg/mL exhibited the most pronounced protective effect (μₑ = 0.0480 h^−1^; Δμ = 0.0959), while isorhamnetin at the same concentration maintained μₑ at 0.0576 h^−1^ (Δμ = 0.0863). These findings suggest that both compounds possess prebiotic potential, with compound **1** showing slightly superior efficacy in enhancing probiotic survival.

### 2.4. Development and Validation of an HPLC Analytical Method

An HPLC method was established for the quantification of the marker compound (compound **1**) in *O. humifusa* leaf extract, using the chromatographic conditions detailed in Section 4. Method validation was carried out in accordance with standard analytical criteria, including assessments of linearity, accuracy, precision, and sensitivity [limit of detection (LOD), limit of quantification (LOQ)].

Linearity was confirmed by generating calibration curves at three concentration levels across a range of 5–500 μg/mL. The resulting calibration equation was *y* = 18,208*x +* 100,460, with an excellent correlation coefficient (*R*^2^ = 0.99), indicating strong linearity. The LOD and LOQ, calculated based on signal-to-noise ratios of 3 and 10, respectively, were determined to be 0.5 μg/mL and 1.9 μg/mL, respectively.

Accuracy was evaluated through recovery experiments by comparing the measured concentrations to their true values. The recovery rate averaged 98.3% with a standard deviation of ± 2.97%, falling within the acceptable range.

Precision was assessed through intra- and inter-day variability tests at three different concentrations of compound **1**. For intra-day precision, six replicate injections were analyzed on the same day, whereas inter-day precision was evaluated by performing six replicates each day over three consecutive days. As summarized in Table 1, the intra-day relative standard deviation (RSD) values ranged from 0.05% to 2.00%, and the inter-day RSDs ranged from 0.07% to 3.01%, all of which are within acceptable limits, demonstrating the robustness and repeatability of the method.

**Figure 4:**
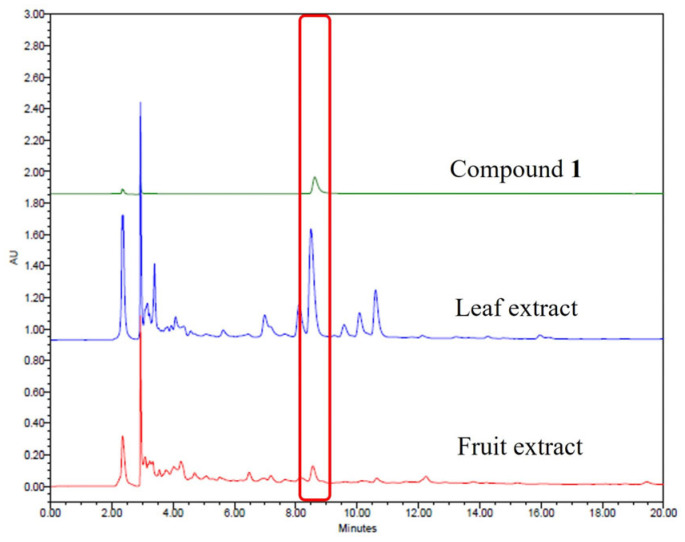
HPLC chromatograms of the ethanol extracts of *O. humifusa* leaves and fruits compared to compound **1**. The peak for compound **1** is marked with a red rectangle in the HPLC chromatogram.

The validated HPLC method was subsequently applied to quantify compound **1** in ethanol extract of *O. humifusa* leaves. Representative chromatograms of compound **1** and the sample extracts are shown in Figure 4. Identification of the isolated compound was based on the comparison of Rf values and UV spectra with those of the crude extract under identical analytical conditions. Quantitative analysis revealed compound **1** to be present at concentrations of 0.33 mg/g in the 80% ethanol extract of the leaves.

### 2.5. Optimization of Extraction Conditions Based on Marker Compound Content

**Figure 5:**
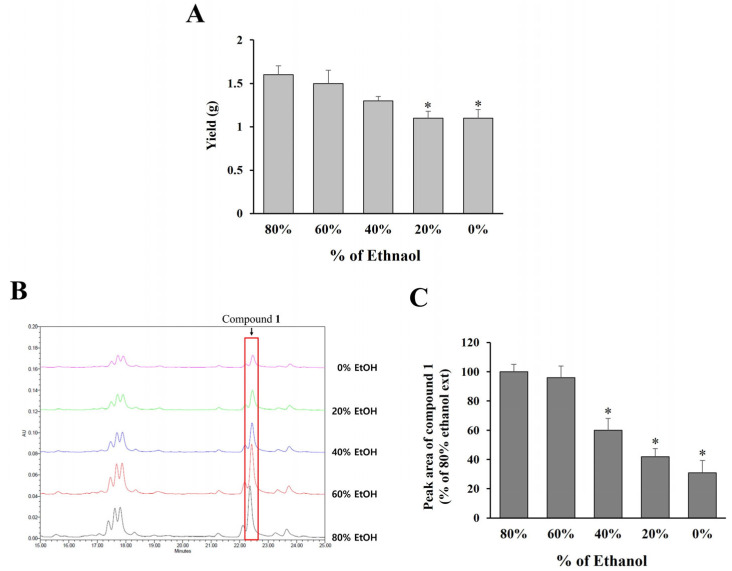
The yields of *O. humifusa* leaf extracts extracted with various percentage of ethanol. (**A**) The amounts of *O. humifusa* leaf extracts extracted with 80–0% ethanol. (**B**) Representative HPLC chromatograms of *O. humifusa* leaf extracts extracted with 80–0% ethanol focused on compound **1**. (**C**) Comparison of the amount of compound **1** in *O. humifusa* leaf extracts extracted with 80–0% ethanol. % Values are displayed relative to 80% ethanol extract. * *p* < 0.05 vs. 80% ethanol extract.

To establish optimal extraction conditions applicable to large-scale production in the industry, *O. humifusa* leaves were extracted using ethanol at concentrations ranging from 0% to 80%, followed by freeze-drying and quantification of compound **1** via HPLC analysis. As shown in Figure 5A, extraction yield increased with higher ethanol concentrations, with 60% and 80% ethanol yielding comparable extract amounts.

Additionally, the content of compound **1** in each extract was assessed by analyzing the peak areas in the HPLC chromatograms (Figure 5B,C). The results demonstrate that both 60% and 80% ethanol extracts contained significantly higher levels of compound **1** compared to extracts obtained using lower ethanol concentrations (0–40%). Considering both extraction efficiency and practical applicability for large-scale production, 60% ethanol was selected as the optimal solvent for extracting *O. humifusa* leaves.

## 3. Discussion

This study aimed to investigate the prebiotic potential of *Opuntia humifusa* (commonly known as Cheon-nyeon-cho) extracts by assessing their ability to promote the viability of *Lactobacillus species*. The findings demonstrated that both water and ethanol extracts of *O. humifusa* leaves and fruits did not significantly affect bacterial growth of *L. paracasei* during early incubation (up to 48 h). However, at 60 h, a clear enhancement in bacterial viability was observed, particularly in the groups treated with the leaf extract. This time-dependent effect suggests that the active constituents in the extracts may require prolonged exposure or reach optimal activity during the stationary phase of bacterial growth.

Several natural compounds have demonstrated prebiotic efficacy by selectively stimulating beneficial gut microbes. Plant-derived extracts from pineapple and *Curcuma xanthorrhiza* have also been reported to stimulate the growth of *Lacticaseibacillus paracasei*, further underscoring their prebiotic potential [10], and turmeric extract has also been demonstrated to enhance the viability of key probiotic strains such as *Lactobacillus rhamnosus* GG and *Bifidobacterium animalis* BB12, suggesting their prebiotic potential may be indirectly supported by their anti-inflammatory properties, which help create a more favorable gut environment for probiotic growth [11]. In addition, soybean oligosaccharides, which include stachyose and raffinose, have been shown to significantly enhance populations of *Bifidobacterium* and *Lactobacillus*, along with improving immune responses in vivo [12]. Similarly, well-known oligosaccharides such as inulin and galacto-oligosaccharides support the proliferation of bifidobacteria and polyclonal growth of *Lactobacillus* spp., while also increasing short-chain fatty acid production and promoting gut health [13,14,15]. These findings suggest that compound **1** from *O. humifusa* may similarly function as a novel prebiotic by promoting probiotic viability and contributing to intestinal health.

The higher efficacy of the leaf extract over the fruit extract in promoting probiotic survival correlates well with the quantification data obtained from HPLC analysis. The leaf extract contained a higher concentration of compound **1**, identified as isorhamnetin 3-*O*-β-*D*-(6-*O*-α-*L*-rhamnosyl)glucoside, a glycosylated flavonol also known as isorhamnetin-3-O-rutinoside. Compound **1** has previously been isolated from various medicinal plants, including *Astragalus annularis* [8], *Calendula officinalis* [16], *Nitraria retusa* leaves [17], and *Averrhoa carambola* [18]. It has demonstrated notable antioxidant and immunomodulatory activities. For instance, extracts containing this compound exhibited strong reactive oxygen species scavenging activity, thereby reducing oxidative stress in cellular systems and animal studies [19]. Additionally, isorhamnetin-3-O-rutinoside was shown to have anti-cancer and immunoregulatory properties in human leukemia (K562) cells [17]. Its favorable intestinal transport characteristics, demonstrated using Caco-2 cell models, also support its potential as a bioavailable nutraceutical ingredient [20]. However, currently, no studies have reported its potential prebiotic effects, indicating a novel functional aspect of this compound as observed in the present study.

Flavonoid glycosides have previously been reported to modulate gut microbial composition by serving as selective growth substrates for beneficial bacteria [21,22]. In the present study, compound **1** was shown to significantly increase the viability of *L. paracasei* KCTC 12576 after 60 h of incubation. Interestingly, its aglycone form, isorhamnetin, also promoted bacterial growth, though the glycosylated form (compound **1**) appeared to offer similar or slightly enhanced effects. This result aligns with previous studies showing that glycoside conjugation can improve the solubility and bioavailability of flavonoids, potentially enhancing their interaction with microbial enzymes and membranes [23].

The development of a validated HPLC method enabled accurate quantification of the marker compound and facilitated the optimization of extraction conditions. Among tested solvent systems, 60% ethanol was determined to be the optimal extraction solvent for maximizing compound **1** yield, offering a practical and efficient method for potential industrial-scale production of a novel natural prebiotic agent.

These findings contribute to the growing body of literature on plant-based prebiotics and suggest that *O. humifusa* leaf extract, particularly its flavonoid content, may be a promising candidate for functional food development aimed at improving gut health.

## 4. Materials and Methods

### 4.1. Preparation of O. humifusa Extracts

Fresh leaves and fruits of *O. humifusa* (1 kg each) were purchased from a local market. Botanical authentication of the plant material was conducted by Prof. So-Young Park, and a voucher specimen (C20210107) was deposited in the Pharmacognosy Laboratory of the College of Pharmacy at Dankook University (Cheonan, Republic of Korea). The plant materials were homogenized using a blender, followed by extraction with either 80% ethanol or distilled water (10 L, 5 times) under constant agitation for 24 h at room temperature. The extracts were filtered and concentrated under vacuum to remove solvents. The concentrated extracts were then lyophilized to obtain ethanol and water extracts, which were stored at −20 °C until use. Ethanol extract yields were 47 g for leaves and 72 g for fruits, while water extract yields were 37 g for leaves and 100 g for fruits.

To determine the optimal extraction conditions, 50 g of *O. humifusa* leaves was extracted with ethanol at concentrations of 0%, 20%, 40%, 60%, and 80% (*v*/*v*) for 24 h at room temperature. The resulting extracts were concentrated and used in subsequent experiments.

### 4.2. Evaluation of Growth-Stimulatory Effects on Lactobacillus Species

The probiotic strains including *Lactobacillus casei* KCTC 14247, *L. paracasei* KCTC 12576, *L. plantarum* KCTC 15194 (each at 1 × 10^9^ colony-forming units per milliliter [CFU/mL]), and *L. acidophilus* LA-5 (Chr. Hansen, Hørsholm, Denmark; 1 × 10^8^ CFU/mL) were inoculated into test tubes containing 10 mL of MRS broth. Various concentrations of ethanol and water extracts of *O. humifusa* or isolated compound(s) were added to the cultures at final concentrations of 0.1% (*v*/*v*), and the mixtures were vortexed briefly to ensure homogenous distribution of the treatment. The cultures were incubated at 37 °C for 12, 36, 48, and 60 h. Bacterial growth was assessed by measuring optical density at 600 nm (OD_600_) using a spectrophotometer (BioTek Instruments, Winooski, VT, USA) and by viable cell count using the standard plate count method. For CFU enumeration, serial dilutions of the cultures were plated on MRS agar and incubated for 48 h at 37 °C before counting. All experiments were performed in triplicate. A control group without extract treatment was included to assess baseline growth conditions.

To better characterize the growth kinetics of *Lactobacillus* spp., OD_600_ values over time were fitted to the modified Gompertz model using nonlinear regression [24]. From this, the specific growth rate (μ) was calculated, providing a more detailed understanding of the effect of the extract on bacterial proliferation.

### 4.3. Isolation of Active Compounds

To remove sugar from the ethanol leaf extract obtained in the previous step, 47 g of the extract was loaded onto a column containing 500 g of Amberlite XAD-4 resin (Sigma-Aldrich, St. Louis, MO, USA). Sequential elution was performed using water (100%), water/methanol (1:1), and methanol (100%). Four fractions (FR1–FR4) were collected, and fraction FR2 (3 g) was further subjected to medium pressure liquid chromatography (MPLC) using a C18 resin cartridge (400 g). The elution was carried out with increasing concentrations of acetonitrile in water (10:90, 25:75, 30:70, 0:100, *v*/*v*), resulting in 16 subfractions (FR2-1 to FR2-16). Fractions FR2-14 and FR2-15 were combined (246.4 mg) and subjected to column chromatography using Sephadex LH-20 resin (GE Healthcare, Uppsala, Sweden). Elution was performed with water, 50% methanol, and then 100% methanol. Three subfractions (FR2-14-1 to FR2-14-3) were obtained, and compound **1** (33.3 mg) was isolated from subfraction FR2-14-2.

The structure of the isolated compound was determined as isorhamnetin 3-*O*-β-D-(6-*O*-α-L-rhamnosyl)glucoside (**1**) through comparative analysis of their 1D (^1^H- and ^13^C-NMR) and 2D (HSQC and HMBC) NMR spectroscopic data with literature values [8,9].

Compound **1** (Isorhamnetin 3-O-β-D-(6-O-α-L-rhamnosyl)glucoside): Yellow powder; ^1^H-NMR(DMSO-*d*_6_, 400 MHz) *δ*H: 7.87 (1H, d, *J* = 2.0 Hz, H-2′), 7.52 (1H, dd, *J* = 2.0, 8.4 Hz, H-5′), 6.92 (1H, d, *J* = 8.4 Hz, H-6′), 6.44 (1H, d, *J* = 2.0 Hz, H-8), 6.21 (1H, d, *J* = 2.0 Hz, H-6), 5.45 (1H, d, *J* = 7.4 Hz, H-1″), 4.42 (1H, d, *J* = 0.9 Hz, H-1′″), 3.91 (3H, s, 3′- OCH_3_), 0.91 (3H, d, *J* = 5.6 Hz, H-6′″). ^13^C-NMR(DMSO-*d*_6_, 100 MHz) *δ*C: 178.2 (C-4), 165.1 (C-7), 162 (C-5), 157.3 (C-2, 9), 150.2 (C-4′), 147.7 (C-3′), 133.8 (C-3), 123.1 (C-6′), 121.9 (C-1′), 116.1 (C-5′), 114.1 (C-2′), 104.8 (C-10), 102.0 (C-1″), 101.8 (C-1′″), 99.6 (C-6), 94.7 (C-8), 77.2 (C-5″), 76.8 (C-3″), 75.1 (C-2″), 72.6 (C-4′″), 71.4 (C-3′″), 71.2 (C-2′″), 70.9 (C-4″), 69.2 (C-5′″), 67.7 (C-6″), 56.5 (3′- OCH_3_), 18.6 (C-6′″).

### 4.4. Acid Hydrolysis of Compound 1

Compound **1** (10.0 mg) was subjected to acid hydrolysis by dissolving it in 5.0 mL of 2 N hydrochloric acid and heating at 90 °C for 2 h. After the reaction, the mixture was partitioned between ethyl acetate and water. The resulting ethyl acetate fraction contained the aglycon isorhamnetin, which was confirmed through comparison with an authentic standard. The aqueous fraction was analyzed by thin layer chromatography (TLC), and the released sugars were identified as rhamnose and glucose based on co-migration with authentic sugar standards by TLC analysis [25,26]. The structure of aglycon isorhamnetin was confirmed through the NMR analysis [27,28].

Compound **2** (Isorhamnetin): Yellow powder; ^1^H NMR (DMSO-*d_6_*, 600 MHz); δ 12.4(1H, s, 5-OH), 7.75 (1H, d, *J* = 2.3 Hz, H-2′), 7.68 (1H, dd, *J* = 2.4, 8.4 Hz, H-6′), 6.94 (1H, d, *J* = 8.4 Hz, H-5′), 6.47 (1H, d, *J* = 2.4 Hz, H-8), 6.19 (1H, d, *J* = 1.9 Hz, H-6), 3.8(3H, s, 3’-OCH_3_); ^13^C NMR ((DMSO-*d_6_*, 150 MHz); δ 175.8 (C-4), 163.9 (C-7), 160.6 (C-5), 156.1 (C-9), 148.7 (C-4′), 147.3 (C-3′), 146.6 (C-2), 135.8 (C-3), 121.9 (C-1′), 121.7 (C-6′), 115.5 (C-5′), 111.7 (C-2′), 103.0 (C-10), 98.2 (C-6), 93.5 (C-8), 55.7 (3′-OCH_3_).

### 4.5. HPLC Method Validation

The HPLC method used for the quantification of compound **1** (isorhamnetin 3-O-β-D-(6-O-α-L-rhamnosyl)glucoside) was validated in accordance with ICH guidelines to ensure its reliability and reproducibility for quality control purposes (Table 2).

The validation parameters included linearity, accuracy, precision (intra-day and inter-day), and quantitative analysis.

- Linearity: To evaluate linearity, a series of standard solutions of compound **1** at three different concentrations (5–500 µg/mL) were prepared and analyzed under the established HPLC conditions. Calibration curves were constructed by plotting the peak area against the corresponding concentrations. The coefficient of determination (R^2^) was calculated to assess the goodness of fit.
- Accuracy (Recovery Test): The accuracy of the method was determined by recovery tests using the standard addition method. Known amounts of compound **1** were spiked into pre-analyzed *O. humifusa* leaf extract samples at three concentration levels (5, 50, and 500 µg/mL). Each sample was analyzed in triplicate, and recovery (%) was calculated using the formula

Recovery (%) = (Detected amount/Added amount) × 100

- Precision: Intra-day precision was assessed by analyzing three replicates of the same sample at three concentration levels within a single day. Inter-day precision was evaluated by performing the same procedure on three consecutive days. The results were expressed as relative standard deviation (RSD, %).
- Quantitative Analysis: The content of compound **1** in *O. humifusa* leaf extracts was determined by external standard calibration using the validated HPLC method. All samples were analyzed in triplicate, and results were expressed as μg of compound **1** per gram of dried extract (μg/g).

### 4.6. Data Analysis

All data were calculated as means ± SDs, and multiple group comparisons were performed using one-way analysis of variance, followed by the Fisher’s least significant difference test (SPSS version 27.0, Armonk, NY, USA). Statistically significant differences between values were considered to be present when the *p* value was below 0.05 (* *p* < 0.05).

## 5. Conclusions

In conclusion, this study provides evidence that *Opuntia humifusa* leaf extracts possess significant prebiotic potential, contributed by the presence of isorhamnetin glycosides. Isorhamnetin 3-*O*-β-*D*-(6-*O*-α-*L*-rhamnosyl)glucoside (compound **1**) was identified as a marker compound that contributes to enhancing the viability of *Lactobacillus paracasei* KCTC 12576. The optimized extraction with 60% ethanol yielded the highest concentration of this marker compound, offering a practical and efficient method for potential industrial-scale production of a novel natural prebiotic agent. These findings suggest that *O. humifusa* leaf extract may serve as a safe, natural, and effective prebiotic ingredient, suitable for incorporation into synbiotic formulations and functional foods aimed at promoting gut health.

## Figures and Tables

**Table 1:** Intra- and inter-day precision values of the HPLC method applied to compound **1**.

Conc (μg/mL)	Intra-Day		Inter-Day	
	Mean ± SD	%RSD	Mean ± SD	%RSD
500	500.03 ± 0.23	0.05	499.93 ± 0.35	0.07
50	50.21 ± 0.87	1.74	50.55 ± 1.09	2.15
5	5.00 ± 0.10	2.00	5.07 ± 0.15	3.01

**Table 2:** Conditions to validate the HPLC analytical method for the *O. humifusa* leaf extract.

Parameter	Method	
Column	Capcell PAK C18 UG80 S-5 (4.6 × 250 mm, 5 μm)	
Mobile phase A	Acetonitrile	
Mobile phase B	Water	
Flow rate	1.0 mL/ min	
Time	Mobile phase A (%)	Mobile phase B (%)
5	20	80
10	24	76
20	26	74
21	100	0

## Data Availability

Data are contained within the article.

## Abbreviations

The following abbreviations are used in this manuscript:

DMSO	Dimethyl Sulfoxide
HMBC	Heteronuclear Multiple Bond Correlation
HPLC	High Performance Liquid Chromatography
HSQC	Heteronuclear Single Quantum Coherence
LOD	Limit of Detection
LOQ	Limit of Quantification
MPLC	Medium Performance Liquid Chromatography
NMR	Nuclear Magnetic Resonance
RSD	Relative Standard Deviation
TLC	Thin Layer Chromatography
